# Stakeholder’s perspectives of postnatal discharge: a qualitative evidence synthesis

**DOI:** 10.1136/bmjgh-2023-011766

**Published:** 2023-08-08

**Authors:** Chloe Mercedes Harvey, Helen Smith, Anayda Portela, Ani Movsisyan

**Affiliations:** 1 Independent Consultant, Bangkok, Thailand; 2 International Health Consulting Services Ltd, Liverpool, UK; 3 Department of Maternal, Newborn, Child and Adolescent Health and Ageing, World Health Organization, Geneva, Switzerland; 4 Institute for Medical Informatics, Biometry and Epidemiology, Ludwig Maximilians University Munich, Munchen, Germany; 5 Pettenkofer School of Public Health, Ludwig Maximilian University of Munich, Munchen, Bayern, Germany

**Keywords:** Maternal health, Qualitative study, Child health, Health policy

## Abstract

**Introduction:**

Discharge preparation prior to leaving a health facility after childbirth offers a critical window of opportunity for women, parents and newborns to receive support for the transition to care at home. However, research suggests that the quality of discharge preparation following childbirth is variable. This review synthesises qualitative evidence on stakeholder perspectives of postnatal discharge.

**Methods:**

We conducted a thematic synthesis of qualitative studies included in a larger published scoping review on discharge preparedness and readiness (reported in accordance with Preferred Reporting Items for Systematic Reviews and Meta-Analyses extension for scoping reviews). For inclusion, in the qualitative evidence synthesis, studies had to have used qualitative methods for data collection and analysis to capture the perspectives of women, parents and health workers. Key characteristics and findings were extracted, and thematic analysis was used to inductively develop a conceptual coding framework.

**Results:**

Of a total of 130 research documents (published research articles and grey literature), six studies met the inclusion criteria; five were conducted in high-income countries, five were published in English and one was published in Swedish. Studies reported on the experiences of women, fathers and midwives with the number of participants ranging from 12 to 324. Nine descriptive themes (findings) were identified. From these, three high-level analytical themes were generated: (1) health workers need support to optimise the postnatal discharge process; (2) the allocated time for, and timing of, discharge is rushed; (3) overlooking women’s and fathers’/partners’ needs leads to feelings of exclusion.

**Conclusions:**

Findings suggest an overall feeling of dissatisfaction among women, parents and midwives with the current provision of discharge preparation. In particular, women and midwives expressed frustration at the lack of time and resources available for ensuring adequate quality of care prior to discharge. The perspectives of included stakeholders indicate a demand for increased focus on the emotional and social needs of women and families during discharge preparation as well as better engagement of fathers and other family members. The qualitative evidence available indicates the likely positive impact of adequate discharge preparation if the identified service and system barriers can be overcome. As the updated WHO recommendations on postnatal care become embedded in country health systems and policies, there may be renewed interest on values, preferences and perspectives at system, service and end-user level.

**WHAT IS ALREADY KNOWN ON THIS TOPIC**

- The quality of discharge preparation following childbirth is variable, substantiated by a recent scoping review of published literature and policy documents, which highlighted the need for comprehensive discharge assessment and education to better identify and meet the needs of women, parents/caregivers and families prior to discharge and identify those who may require additional support.
- A published qualitative synthesis on postnatal care shows that women and parents report feeling unprepared for the postnatal period, lacking the knowledge to care for themselves and their newborns.

**WHAT THIS STUDY ADDS**

- This qualitative evidence synthesis addresses a knowledge gap on stakeholder’s perspectives (women, fathers and health workers) of postnatal discharge.
- Findings confirm an overall feeling of dissatisfaction among women, fathers and midwives with the current provision of discharge preparation.
- Taking into consideration the perspectives of women, fathers and midwives, the available qualitative evidence suggests that there is demand to include the emotional and social needs of women and families during discharge preparation.

**HOW THIS STUDY MIGHT AFFECT RESEARCH, PRACTICE OR POLICY**

- This study highlights the importance of discharge preparation as a critical opportunity to ensure the woman, parents and newborn receive support for the transition to care in the home.
- Findings of this review support the updated WHO postnatal care recommendations, which include broadened criteria to be assessed at discharge, such as the skills and confidence of the woman to care for herself and of parents to care for the newborn as well as emotional and social needs.
- As the updated WHO recommendations on postnatal care become embedded in country health systems and policies, there may be renewed interest on values, preferences and perspectives at system, service and end-user level.

## Introduction

The postnatal period is a time of significant transition for women and newborns, comprising several biological, psychological and social adjustments for women as they recover from childbirth and navigate motherhood,^1 2^ as well for their families, as self-identities are redefined.^3^ The early postnatal period also represents one of the riskiest periods for women and newborns,^4^ with globally, an estimated 800 women dying daily due to complications of pregnancy and childbirth,^5^ the majority occurring during birth or within 24 hours of birth. As a result, global maternal and newborn health strategies have focused on increasing the rate of facility birth to ensure that women and newborns have access to skilled birth attendance,^4^ to minimise the risk of morbidity and mortality from complications. Although efforts to positively encourage facility births have been successful in many countries, this has not corresponded with expected improvements in health outcomes in others, particularly in countries in Africa and Asia.^6^

Care received in the facility in the period immediately following birth is critical for women’s and newborns’ survival and provides an opportunity to support the transition from care in the facility to providing care at home.^7^ A positive postnatal experience, where women feel supported to adapt to their new role as a mother and build self-confidence in caring for their newborn while navigating the physical and emotional challenges following birth, was highlighted by women to be important in a recent review of qualitative evidence.^8^ However, the quality of care provided in facilities is variable and postnatal care often receives less attention from health workers than antenatal and intrapartum care.^9 10^ Women report feeling unprepared for the postnatal period, lacking the knowledge to care for themselves and their newborns, while at the same time feeling overwhelmed with exhaustion and emotional stress following childbirth.^8 11 12^ When women and newborns are not ready for postnatal discharge, there is the potential for poorer health outcomes for women and their newborns, risk of readmission and consequent increased financial burden on the health system and on families.^13 14^

The WHO recommends all women and newborns remain in the facility for at least 24 hours after non-complicated vaginal birth.^15^ In addition to providing continuous care to allow sufficient time to detect potential complications and provide adequate education to women and parents, it is proposed that this time also be available for providing support and education to women and parents to facilitate the transition to the home.

A scoping review of global policies, guidelines and literature on discharge preparedness and readiness after birth,^9^ found gaps in current policies and identified a need for more comprehensive discharge assessment and education to prepare women, parents/caregivers and families for discharge. While the broader scoping review mapped all available literature including policies, guidance and peer-review literature on discharge preparation and readiness, this paper specifically focuses on the qualitative studies that report on stakeholder’s perspectives (women’s, parents’^1^ and health workers’) of postnatal discharge, identified through the search conducted for the scoping review. Previous qualitative evidence syntheses have summarised women’s and health workers’ perspectives on postnatal care,^8 16 17^ but none has specifically explored perspectives on discharge preparation.

## Methods

We conducted a synthesis^18^ of the qualitative studies derived from the larger scoping review that was conducted and reported in accordance with the Preferred Reporting Items for Systematic Reviews and Meta-Analyses (PRISMA) extension for scoping reviews. The methods guide for the larger scoping review was registered on protocols.io.^19^

### Search strategy and selection criteria

The description of the search strategy and selection criteria is detailed in the published manuscript of the larger scoping review.^9^ Fourteen electronic databases were searched to locate published research for the original scoping review^9^; see online supplemental file 1 for, an example, search strategy. Components of the search strategy pertinent to this qualitative evidence synthesis (QES) are outlined here.

10.1136/bmjgh-2023-011766.supp1 Supplementary data

For inclusion, studies had to use qualitative methods for data collection (eg, in-depth or semistructured interviews, focus group discussions, observation, document analysis, open-ended survey responses) and analysis (eg, thematic analysis, grounded theory, thematic framework analysis). Studies were excluded if they did not meet these criteria, or if the methods were not reported (table 1). We used the PerSPectif framework to formulate inclusion/exclusion criteria.^20^

**Table 1:** Inclusion and exclusion criteria relevant to the QES

	Inclusion criteria	Exclusion criteria
Perspective	Healthy women who have given birth, newborns and fathers/parents/caregivers/family members Midwives/nurses/other health workers or providers of maternity care in a facility	Women who have given birth, with identified clinical complications; other participant groups, unrelated to postnatal care Women, newborns and parents/caregivers/family members after a home birth
Setting	Any country	
Phenomenon of interest	Discharge preparation or discharge readiness after facility birth	Discharge preparation or readiness for other health causes
Environment	Facilities offering maternity care	
Timing	The period after childbirth until discharge from the facility	
Findings	Perspectives on the postnatal discharge process	
Type of document	Research studies reporting qualitative data collection and analysis methods (including mixed-methods studies)	Conference abstracts
Language	Any language	
Date limits	From 2000, when studies on the effect of postnatal discharge began to appear in the literature, to 2020	Prior to 2000

QES, qualitative evidence synthesis.

Studies conducted in low-income, middle-income and high-income countries and those written in any language were considered for inclusion. Conference abstracts were not included in the current review on the basis that they provide insufficient information on the methods used and the full findings are usually not reported.

**Figure 1:**
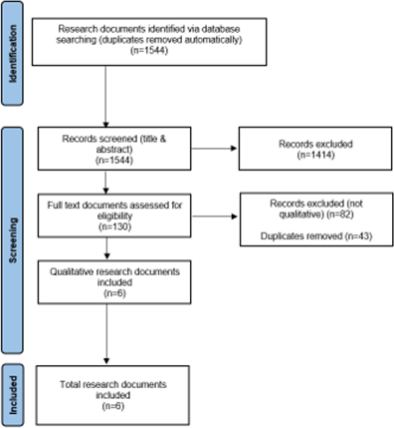
Flow diagram of screening and selection process.

Screening and selection decisions are documented in the PRISMA flowchart (figure 1).

### Quality assessment of the primary studies

The quality of included studies was appraised using the Critical Appraisal Skills Programme (CASP) tool for qualitative studies,^21^ designed to assess three broad areas: whether the results of the study are valid, what the results are and whether they can be applied locally. The CASP checklist was converted to an Excel spreadsheet where for each domain, reviewer comments were recorded to explain reasoning for the rating and to aid further discussion among all authors. Based on the rating within each domain, an overall rating was assigned to each study, ranging from ‘no or very minor concerns’ to ‘moderate concerns’. Although quality assessment of individual studies was not used as a basis for exclusion, it contributed to the assessment of confidence in the review findings.

CMH conducted the initial quality assessment of studies, which was cross-checked by both HS and AP to ensure consistency.

### Data extraction and synthesis

An MS Excel sheet was used to extract basic information about the studies, such as first author, date of publication, publication language, study context, study design, study aim, participants, analysis method and number of participants as well as findings on stakeholders’ perspectives (table 2). For the one research study that was in Swedish, a native Swedish speaker was contacted and extracted the basic information and main findings.

**Table 2:** Characteristics of included studies

Citation	Country	Aim	Study design	Participants	Stakeholder’s perspective(s)	Quality rating (CASP)
Dol *et al*^23^	Tanzania	To explore the experience of newborn care discharge education at a national hospital from the perspective of mothers and nurse midwives.	Qualitative; descriptive In-depth semi-structured interviews	Mothers who had given birth within the past 72 hours (n=8) Nurse midwives working on the postnatal and labour ward (n=8)	Women, Midwives	Minor concerns
George^29^	USA	To examine the experiences of first-time mothers following discharge from the hospital after vaginal delivery	Qualitative; grounded theory In-depth interviews	Primiparous mothers who had an uncomplicated vaginal birth (n=21)	Women	Moderate concerns
Haith-Cooper *et al*^31^	England	To explore the perceptions of women and senior student midwives related to the postnatal hospital discharge process and maternal sepsis prevention advice	Qualitative; phenomenology Focus group discussions	Women who had given birth in the past year (n=9), vulnerable migrant women who had given birth in the past year (n=5), third year student midwives (n=9)	Women, Student Midwives	No or very minor concerns
Kanotra *et al*^27^	USA	To identify challenges that women face 2–9 months postpartum using free text comments gathered by the Pregnancy Risk Assessment Monitoring System (PRAMS).	Qualitative; codebook analysis of free text comment data	Women respondents to the PRAMS survey from 10 states, who provided free-text comments on postpartum concerns (n=324)	Women	Minor concerns
Persson and Dykes^30^	Sweden	To investigate factors that influence the experience of mothers and fathers when they have chosen to return home, earlier than is the normal routine, following the birth of their baby.	Qualitative; grounded theory Open interviews	Mothers (n=6) and fathers (n=6) who left maternity/family ward within 26 hours of birth	Women, Fathers	No or very minor concerns
Svensson^28^	Sweden	To investigate first-time mothers’ experiences of support from maternity ward related to early hospital discharge after childbirth	Questionnaire Qualitative content analysis	First-time mothers (n=15)	Women	Moderate concerns

CASP, Critical Appraisal Skills Programme.

Findings were extracted from each included study by one reviewer (CMH) and cross-checked by the others (HS, AP and AM). The findings were extracted verbatim into a preprepared spreadsheet, which organised the findings by the type of stakeholder expressing a perspective (women/fathers, family members and other caregivers/health workers). Thematic analysis^22^ was used to inductively develop a conceptual coding framework using a study, which most accurately reflected the overall focus of the review as an index study.^23^ This framework was iteratively adjusted as findings from all the included papers were coded and mapped to the framework in a spreadsheet. The extracted findings were also disaggregated by the stakeholder who expressed them. Once coding was complete, one author (CMH) regrouped coded extracts into preliminary themes, which were discussed by all authors to ensure relevance and clarify meaning; any disagreements were discussed and resolved through consensus. Throughout the analysis, evidence was sought to confirm and also disconfirm the emerging themes. Following the assessment of confidence in the review findings (see below), the descriptive themes were further grouped into higher level analytical themes, which captured the main narratives emerging from the studies.

### Assessing confidence in the QES findings

The confidence in the review findings (ie, the descriptive themes) was assessed using the GRADE-CERQual approach based on four components: methodological limitations, coherence, adequacy of data, and relevance.^24^ Each review finding was assessed to have ‘no or very minor concerns’, ‘minor concerns’, ‘moderate concerns’, or ‘serious concern’ in relation to these components based on the contributing body of evidence. An overall rating was then developed for each review finding in light of the assessment across the four components. The final confidence rating was classified into one of the following categories: ‘high’, ‘moderate’, ‘low’ or ‘very low’. These represent the extent to which the review findings are reasonable representations of the phenomenon of interest (ie, perspectives of discharge preparation).^25^ The GRADE-CERQual assessment^26^ was initially conducted by AM and further discussed among the author team documenting all judgements and decisions (see table 3). The performance of each review finding against each of the four GRADE-CERQual components is provided in online supplemental file 2.

10.1136/bmjgh-2023-011766.supp2 Supplementary data

**Table 3:** Framework of descriptive and analytical themes with explanation of confidence in the evidence assessment

Number	Descriptive theme (stakeholder’s perspective represented)	Studies contributing to the descriptive theme	GRADE-CERQual assessment of confidence	Explanation of confidence in the evidence assessment	Analytical theme
1	Diversification of teaching methods needed	Dol^23^; Persson^30^; Kanotra^27^; Svensson^28^	Moderate	This finding was graded as moderate confidence because of moderate concerns regarding coherence and adequacy and minor concerns regarding methodological limitations.	1.Health workers need support to optimise the postnatal discharge process
	Postnatal care education often lacks the opportunity for mothers and parents to practice care with the support of a health worker, especially for breastfeeding support. Views expressed identified a need for diversification of teaching methods, including varied media. (Women/Midwives)				
2	Need for standardised and comprehensive procedures	Dol^23^; Haith-Cooper^31^	Moderate	This finding was graded as moderate confidence because of moderate concerns regarding adequacy and minor concerns regarding methodological limitations.	
	Midwives expressed the importance of delivering a consistent quality of care, which requires clear guidelines on the topics to be covered during postnatal discharge education. Women reported that important information was sometimes omitted (eg, sepsis danger signs) (Women/Midwives)				
3	Need for midwife training on postnatal care education	Dol^23^	Moderate	This finding was graded as moderate confidence because of serious concerns regarding adequacy and minor concerns regarding methodological limitations.	
	Midwives expressed a need for in-depth training on delivering postnatal care education, especially for care of preterm babies. (Midwives)				
4	Assumed maternal knowledge	Dol^23^; George^29^	Low	This finding was graded as low confidence because of serious concerns regarding adequacy and moderate concerns regarding methodological limitations.	
	Women expressed that there is a perception that women already possess the knowledge to care for themselves or their newborn if they are multiparous, or that information has already been communicated during antenatal care. Multiparous women expressed that they would still prefer to receive postnatal education. Midwives also expressed that they expected women to have already received some education prior to delivery. (Women/Midwives)				
5	Rushed discharge process	Dol^23^; George^29^; Haith-Cooper^31^	Low	This finding was graded as low confidence because of serious concerns regarding adequacy, moderate concerns regarding relevance, and minor concerns regarding methodological limitations.	2.The allocated time for, and timing of, discharge is rushed
	Too much information is provided; there are time limitations and health workforce shortages. (Women/Midwives)				
6	Importance of women/parent involvement in the discharge process	Persson^30^; Kanotra^27^; Svensson^28^	Moderate	This finding was graded as moderate confidence because of serious concerns regarding adequacy.	
	Parents expressed they would like more autonomy over the timing and readiness for hospital discharge, which should be reinforced by the midwife’s empowering behaviour. (Women/Fathers)				
7	Care for the woman is often overlooked	Dol^23^; George^29^; Kanotra^27^; Svensson^28^	Moderate	This finding was graded as moderate confidence because of moderate concerns regarding adequacy and methodological limitations.	3. Overlooking women’s and father’s/partner’s needs leads to feelings of exclusion
	Women expressed the main focus is on the newborn and women need information about their own self-care and expectations. The pain experienced by women is sometimes overlooked. (Women)				
8	Importance of involving fathers/other family members	Dol^23^; Persson^30^; Kanotra^27^; Svensson^28^	Moderate	This finding was graded as moderate confidence because of moderate concerns regarding adequacy and minor concerns regarding methodological limitations.	
	Women expressed a need for increased involvement of fathers/partners/other family members in postnatal education and father’s expressed affinity within the family when they were able to participate. Midwives expressed a need for training on how to engage mothers, fathers and families. The importance of providing information about postpartum depression to parents (especially other family members) was highlighted by women. (Women/Fathers/Midwives)				
9	Socioeconomic, cultural and language barriers	Dol^23^; Haith-Cooper^31^; Kanotra^27^	Moderate	This finding was graded as moderate confidence because of moderate concerns regarding adequacy and minor concerns regarding methodological limitations.	
	Societal norms impact on how postnatal education is received. Information is unavailable in different languages. Length of stay may be influenced by financial/insurance constraints. (Women/Midwives)				

### Patient and public engagement

Patients and the public were not directly involved in the QES reported in this paper. However, patient consumer and health representatives were involved in identifying the question for the larger published scoping review,^9^ as members of the WHO guideline development group for which the scoping review was conducted.

## Results

A total of 130 research documents were identified for full-text screening in the literature search for the larger scoping review. Only six of these original 130 research documents were qualitative and also met our inclusion criteria (figure 1). The characteristics and quality appraisal of included papers are summarised in table 2. Studies were conducted in Sweden (n=2), the USA (n=2), England (n=1) and the United Republic of Tanzania (n=1). Five were published in English and one in Swedish. Four out of the six studies used qualitative data collection techniques such as interviews and focus group discussions, while two studies conducted content analysis,^27 28^ one analysing free-text comment data from a self-administered survey^27^ and another analysing free-text responses from a written questionnaire.^28^ Two papers reported on the experiences of primiparous mothers (a woman who has given birth for the first time) and fathers,^28 29^ two on the experience of early hospital discharge from the perspective of women and fathers,^28 30^ and two studies also collected data on the perspectives of midwives in addition to the perspectives of women and other family members.^23 31^ The number of participants in the included studies ranged from 12 to 324 (table 2).

We generated nine descriptive themes (review findings), and from these, we generated three higher level analytical themes (table 3): (*1) Health workers need support to optimise the postnatal discharge process; (2) the allocated time for, and timing of, discharge is rushed; (3) overlooking women’s and fathers’/partners’ needs leads to feelings of exclusion*.

### Analytical theme 1: health workers need support to optimise the postnatal discharge process

Analysis revealed a perceived need from both the perspectives of midwives and women, to strengthen the postnatal discharge process through ensuring health workers have the necessary resources and capacity to deliver the best quality of care. Although the perspectives of women and midwives contributed to this analytical theme, women in one of the studies referred to other health workers such as doctors and nurses.^27^ Four descriptive findings contributed to this theme; the CERQual rating indicates moderate confidence in three of these, and low confidence in the fourth.

#### Finding 1: diversification of postnatal care teaching methods needed (perspectives of women and midwives) (moderate confidence)

Opportunities for women and caregivers to practice care with the support of a health worker was perceived by midwives and women as an essential component of postnatal care education for improving maternal confidence.^23 27 28 30^ However, women in three of the studies indicated a lack of opportunities to practice newborn care practices that were taught by health workers, and in particular, expressed a clear need for increased breastfeeding support in the hospital, accompanied by demonstration.

Please try to give new mothers in the hospital more lessons on breast feeding. I (myself) would have loved to have been shown how to latch the baby onto my breast. I didn’t know how and was too scared to try alone. (Kanotra 2007; USA)

Some women reported that they would have liked to have received more education from midwives about general newborn care, such as advice on holding, bathing and clothing the baby, aspects of which tended to be overlooked, especially among multiparous women. However, there seemed to be some divergence on the preferred strategies for receiving such information among respondents. Some women indicated a preference for written educational resources such as pamphlets and leaflets that could be taken home, while others expressed a desire for in-person parenting classes delivered by a doctor or nurse prior to discharge. In one study,^28^ women expressed a desire to receive information both in writing and orally during pregnancy and antenatal care visits, to help them prepare for the postnatal period.

#### Finding 2: need for standardised and comprehensive procedures (perspectives of women and midwives) (moderate confidence)

The two studies that included perspectives of midwives indicated a need for standardised and comprehensive procedures for the postnatal discharge process, to ensure consistent quality of care including clear guidelines on the topics to be covered during discharge education.^23 31^

We use the guidelines from the Ministry of Health in which they have outlined standards of postnatal care but not specifically [postnatal] health education. They mention health education and topics that you may touch on, but it is not a guideline on how to give postnatal education (Dol 2019; Tanzania)

A lack of consistency in the information provided to women during discharge education meant that some women missed out on receiving essential information altogether, or information was received too late for it to be useful or potentially lifesaving. In one study that explored the perceptions of women and senior student midwives on hospital postnatal discharge and sepsis advice, women reported receiving insufficient and inconsistent information about sepsis prevention, including divergence in the terminology used and the resources provided.^31^

But I think I didn’t know that sepsis was, like, such a big killer and things like that, so I think if they opened with that, it would stick more because you’re more ‘oh, this is a real problem*’* (Haith-Cooper 2018; England)

Moreover, it was noted that the timing of sepsis advice meant that women sometimes received this critical information after discharge from the facility during a postnatal home visit, at which point, it may be too little, too late.^31^

Nurse midwives interviewed in a national hospital in Dar es Salaam, Tanzania noted a lack of clear guidance on the topics to be covered during discharge education with women and further suggested that developing user-friendly, standardised guidelines for postnatal clinical care as well as postnatal education for mothers would be beneficial.^23^

If we were to have a statement of practice on the wall, that would make it very easy (Dol 2019; Tanzania)

#### Finding 3: need for midwife training on postnatal care education (perspectives of midwives) (moderate confidence)

Midwives from the same study conducted in Tanzania also expressed a need for in-depth training on delivering postnatal care education, especially specialised training on care of preterm babies.^23^

All we have is general education on how to take care of a baby but not specific education like how to take care of a premature baby (Dol 2019; Tanzania).

#### Finding 4: assumed maternal knowledge (perspectives of women and midwives) (low confidence)

In two of the identified studies, women felt that they had a knowledge deficit^23 29^ and reported that it was assumed by midwives that they already possessed the knowledge to care for themselves or their newborn if they were multiparous, or that this information had already been provided during antenatal care.^23^ Similarly, midwives also reported that there is an expectation among midwives that women have already received education during pregnancy and expressed that perhaps this is a more suitable time for this education to be delivered.^23^

I think lack of education contributes a lot to that. Education should be given at the [antenatal] clinic in order for the woman to understand what to do once they go home with a baby (Dol 2019; Tanzania).

The resultant knowledge deficit left mothers feeling unprepared for the transition home and without clear expectations for the early postpartum period, which for some mothers led to information-seeking behaviour among family and friends.^29^

### Analytical theme 2: the allocated time for, and timing of, discharge is rushed

All six included studies referred to the timing of discharge.^23 28–31^ Stakeholders (mothers, fathers and midwives) shared insights into the timing of postnatal hospital discharge, from their perceived readiness, to the influential role that health workers played in facilitating a positive discharge experience. Two descriptive findings contributed to this theme; there is overall low confidence in one finding, and moderate confidence in the other.

#### Finding 5: rushed discharge process (perspectives of women and midwives) (low confidence)

The feeling that the discharge process was rushed a prominent theme that emerged in three studies,^23 29 31^ which left women and families feeling overwhelmed and underprepared for the transition home. Women cited being overloaded with information during discharge education, which coupled with insufficient practical teaching time, made it difficult to retain information.

Too much information from too many sources (George 2005; USA)

Some accounts indicated that women felt they were being rushed through the discharge process because the bed was needed for the next woman.^31^

It felt like it was quite standard that everybody’s out at a certain time ready for the next lot to come, that’s…. I felt like it was like a hotel checkout, everybody was flying out (Haith-Cooper 2018; England).

Midwives also acknowledged that the discharge process is often rushed due to their heavy workloads and multiple responsibilities in providing clinical care to mothers and newborns, as well as education.^23 31^

There’s so much, and when you’re rushing doing a discharge and you’re trying to get TTOs [‘to take out’, i.e. hospital discharge prescriptions] together and all the rest of it (Haith-Cooper 2018; England)

In one study, nurse midwives expressed that their lack of time for discharge processes was a result of having a low ratio of nurse midwives to mother–baby pairs, which is further exacerbated by staff shortages.^23^

I think to make it easier, there should be enough staff to take care of all of the mothers. You may find there are ten patients and I have to give education to all of them. It becomes difficult for me to take enough time as is required to give sufficient education to all of them… (Dol 2019; Tanzania)

Conversely, some women expressed that they preferred an accelerated discharge process because they wanted to return home quickly.^31^

I was rushing it because I just wanted to go…[I answered] ‘Yes’ to every question, ‘okay, brilliant, go, go, go,’ but I don’t think the person doing it was rushing at all, so that was good (Haith-Cooper, 2018; England).

#### Finding 6: importance of parental involvement in the discharge process (perspectives of women and fathers) (moderate confidence)

The need for parental involvement in the discharge process and decisions was identified in two studies, in which parents expressed both positive and negative experiences of the discharge process, in particular, the level of decision-making power they had over the timing of discharge.^27 28 30^

The positive empowering behaviour of midwives in a Swedish hospital was highlighted as an important factor for helping to reinforce a sense of security and safety in parents, allowing parents to feel ownership over the birth and the decisions thereafter.^30^ The midwives’ ‘attitude of competence’ and ‘unstressed professionality’ also appeared instrumental for supporting parental self-confidence in caring for the newborn.^30^

*…*that’s how I experienced it and later here at home as well, this is mine, this is a choice we have made by taking him home….It was built up from the beginning, just that, that we felt confidence in them, the calm, how they dealt with it, it was fantastically meaningful (Persson, 2002; Sweden).

Our findings suggested that discussion with parents over the timing of postnatal hospital discharge is invaluable for women and other family members, to account for the differential needs and preferences following birth. While some mothers expressed that they were grateful for early discharge^2^ due to a lack of peace and quiet in the birth clinic^30^ or because their partner was not permitted to stay with them,^28^ others expressed a perceived need for an extended facility stay, requiring more time to recuperate, both physically and emotionally.^27^

There is this pressure to get out of the hospital and this attitude that you’re a lazy or weak person if you are exhausted and not ready. I had to be forceful to make the hospital and doctors ‘allow’ me to stay in 3 days after a c-section (Kanotra, 2007; USA).

### Analytical theme 3: overlooking women’s and fathers’/partners’ needs leads to feelings of exclusion

All six included studies mentioned factors that could lead to feelings of exclusion among women and fathers or partners. Three descriptive findings contributed to this theme, and all were rated moderate in the CERQual assessment.

#### Finding 7: care for the woman is often overlooked (perspectives of women) (moderate confidence)

The depth of pain and discomfort experienced by women were unexpected,^29^ and yet education included little information on self-care practices during the early postpartum period, which left women with questions about how to look after themselves.^23 27–29^

Many were surprised by the pain, describing it as ‘more than expected’ or unanticipated (George 2005; USA).

This was also noted by midwives who acknowledged that sometimes the pain experienced by mothers, meant that they were not in a good physical condition to receive education and retain information.^23^ In one study, in particular,^28^ women remarked that due to the main focus on the baby, there was insufficient support for breast feeding.

Furthermore, in one study, both mothers and fathers indicated that insufficient information or awareness-raising was provided about postpartum depression.^27^

I believe that health care workers should talk to mothers about depression that can occur after delivery. My depression was very overwhelming…I need more emotional care than physical… (Kanotra, 2007; USA)

#### Finding 8: importance of involving fathers/other family members (perspectives of women, fathers and midwives) (moderate confidence)

Increasing the involvement of fathers, partners and other family members in education was a theme that emerged in four of the studies.^23 27 28 30^ When fathers were effectively engaged in the discharge process, they expressed a sense of participation, nurturing a sense of closeness and attachment within the family.^30^

*….*It [responsibility of care] was built up from the beginning, just that, that we felt confidence in them [the midwives], the calm, how they dealt with it, it was fantastically meaningful (Persson 2002; Sweden).

Women reported that fathers felt excluded during education sessions and missed out on receiving vital information about the risks of postpartum depression and how to respond if their partner displays sign.^27^

In terms of my experience with depression during my recent pregnancy, I would suggest more inclusion of the fathers in any behaviour/mental health counselling of moms. I always felt everything was on my shoulders, my partner always felt left out during appointments… (Kanotra, 2007; USA)

In the context of early hospital discharge, women remarked that inclusion of partners and other family members was particularly key to a smooth transition home.^28^ This was also acknowledged by nurse midwives in one study,^23^ who indicated a desire to receive training on how to effectively engage mothers and other family members in postnatal education.

#### Finding 9: socioeconomic, cultural and language barriers (perspectives of women and midwives) (moderate confidence)

Our findings suggest that some women and their family members experience exclusion during the discharge process if they face socioeconomic, cultural or language barriers and that there is often little done on the part of the health provider to accommodate this.^23 27 31^

Student midwives in one of the studies expressed that a lack of resources provided in different languages or interpreters to translate verbal information meant that there was a heavy reliance on relatives of the mother to interpret information during discharge.^31^ As a result, the consistency and accuracy of translated information were a concern.

It’s normally a younger sister or, like, her equivalent. I’ve seen they…mostly they would pass the information onto female relatives and it tends to be sort of like the second….generation, so a female relative, so they maybe were born in the UK and are native speakers…you can get the closest to that you can get really. (Haith-Cooper, 2018; USA)

Furthermore, sociocultural factors appeared to impact on how postnatal education was received. Nurse-midwives in a hospital in Tanzania observed that women often faced challenges to put the teaching provided at the hospital into practice, due to their own beliefs or because of the pressure of family members.^23^ This was especially apparent when providing advice about feeding practices or the giving of colostrum.

Some of the mothers after giving birth, go to live with their aunts or mother in law. When she reaches there, the aunt might instruct her not to breastfeed her baby with colostrum claiming that it is dirty and not good for the baby (….). These are the challenges that the society must be given education about. And these are things that they have been told at the hospital but the society they live in contradicts our teaching. (Dol, 2019; Tanzania)

Financial barriers or insufficient insurance coverage also posed a significant barrier for an optimal discharge experience for some women whose perceived need for an extended hospital stay could not be fulfilled.^27^

## Discussion

This review of qualitative evidence, published between 2000 and 2020, synthesises the perspectives of different stakeholders, namely women, fathers and midwives, of the processes related to discharge of women and newborns after a facility birth. The review focuses on the perspectives of healthy women and newborns from the general population and does not incorporate the views of those with identified clinical complications.

Findings suggest general feelings of dissatisfaction among women and sometimes fathers about the discharge experience, which is also supported by the perspectives of midwives in some studies, who recognise the shortcomings of health facilities to provide adequate discharge preparation despite there being a demand and desire to provide this. From the nine descriptive themes which were derived during thematic analysis, three higher level analytical themes were established: (*1) health workers need support to optimise the postnatal discharge process, (2) the allocated time for, and timing of, discharge is rushed and (3) overlooking women’s and fathers’/partners’ needs leads to feelings of exclusion*.

This review revealed a perceived need among women for more opportunities to receive orientations about self-care and care of the newborn prior to discharge and that this education incorporates support from health workers to practice care, especially when it comes to breast feeding, bathing and holding the newborn. However, women’s preferences diverged on preferred teaching methods for postnatal education, supporting the suggestions of other studies, which advocate for an individualised approach to meet the unique needs of each mother–infant dyad.^13^ Although midwives in one study expressed a desire to dedicate more time to education sessions,^23^ a lack of support materials for health workers, limited staffing and overburdened health services seem to pose barriers for ensuring consistent and adequate quality of care during the stay in the facility prior to discharge. A lack of continuity in the care of women after birth such as follow-up with the same midwife was also previously highlighted by both women and obstetric clinicians in a large urban teaching hospital in the USA, where both expressed frustration at not being able to form a trusting relationship with the other, hindering women from feeling comfortable to ask questions about their care or postpartum concerns.^11^ In this same study, obstetric clinicians also reported that they felt they lacked time or skills to provide support for psychosocial issues, reinforcing the finding that care prior to postnatal discharge often tends to overlook the emotional and social needs of women and families, focusing primarily on clinical care. Our larger scoping review also found that guidelines and policies were less likely to mention assessment of skills and confidence of the woman to take care of herself, and of skills and confidence of parents, caregivers and families to take care of the newborn, and of the woman’s emotional well-being, whereas the included research documents did. The research literature also indicated the importance of assessing the home environment and other social factors that may affect care in the home and care seeking. The updated WHO recommendations for a positive postnatal experience include broadened criteria to be assessed at discharge, recognising the importance of skills and confidence of the woman, her emotional and social needs, the need to ensure that health workers are skilled to provide discharge support and that linkages to the system are made for follow-up care in case needs are identified.^15^

Based on our findings, for women to feel empowered about their birthing experience and develop the confidence needed to take care of themselves and their newborn, parental involvement is needed for postnatal discharge, as has also been raised elsewhere.^32^ Although timing of postnatal discharge is something that featured in every study included in this review, there was some divergence among stakeholders on when this should happen and how this should be decided. Women and midwives alike, reinforced the narrative of a rushed discharge process,^23 29 31^ that left women feeling unprepared for the transition home with some women commenting on their desire for an extended facility stay to allow them to recover both physically and emotionally,^27^ and learn the skills and knowledge required to take of themselves and their newborn. As length of facility stay has gradually decreased since the 1950s in high-income countries,^33^ concern for early postnatal discharge and its potential consequences on adverse morbidity outcomes and increased readmissions for both mothers and infants has previously been discussed in two systematic reviews.^34 35^ The recently released WHO guidelines for postnatal care continue to recommend a 24-hour length of stay after a vaginal birth, depending on the woman’s and newborn’s needs.^15^ The findings of this QES confirm that stakeholders would like this time to be used well to ensure women and families feel prepared for transitioning to care in the home. However, there has been less focus on understanding what this means for the physical and emotional readiness of women themselves. The finding that some women prefer an earlier discharge than currently stipulated in their context, also supports a shift towards allowing women and parents to participate in discussions about timing of discharge, although this seems to be at odds with the competing pressure on hospital beds, limited staffing and capacity that are often faced by health facilities.

In terms of the content of care provided prior to postnatal discharge, our review suggested that women may feel that information about their own self-care and expectations is often overlooked, with focus on care of the newborn.^27 29^ In particular, the depth of pain and discomfort experienced by women was unexpected,^23 29^ something which was also noted by women in a qualitative study on perceptions of the postpartum experience, in which mothers expressed they were not prepared for the symptoms they experienced and wish that their healthcare providers had been forthcoming, regardless of how common the symptom.^11^ Furthermore, this QES reported an observed disconnect between the concerns of women, which mainly centred around how to manage their symptoms to maintain daily functioning and midwives who were primarily concerned with checking for danger signs, such as infection and bleeding. Similarly, our recently published scoping review, which mapped discharge readiness criteria from policy documents, also indicated that assessment of maternal and newborn physiological stability tended to be the main criteria used to assess discharge readiness, with assessment of the maternal condition often overlooked.^9^

Although only one of the included studies in this review reported on the perspectives of men/fathers themselves,^30^ the importance of involving men and other family members in postnatal education prior to discharge emerged as a common theme, which was also expressed by women and midwives who perceived a need for more training to engage family members.^23 27 30^ Fathers expressed a positive experience of the discharge process when they were effectively engaged as it fostered a sense of participation.^30^ Other studies on the broader postnatal period suggest that fathers desire to be involved in providing care to the newborn as early as possible following birth, which can also positively impact the woman’s physical and emotional well-being.^36 37^ A recent qualitative review highlights important implementation considerations but confirms that men would like to be better engaged in maternal and newborn health, but limited health system capacity hinders their engagement.^38^ Making the discharge process inclusive of men and other family members^39^ is perhaps a preferable first step to ensuring the readiness of the household to support the women in caring for herself and to support the care of the newborn.

Finally, the qualitative literature drew attention to additional barriers experienced by women as a result of socioeconomic disadvantage (especially relevant for contexts with a private health insurance-based system), cultural or language barriers.^27 31^ Similar themes have also emerged in a recent review of research conducted in the United Kingdom,^40^ in which ethnic minority women reported a negative postnatal experience in hospital due to communication barriers and stereotyping by health workers as well as limited adjustments to care to accommodate for cultural traditions around rest and expected duration of hospital stay.

Despite the comprehensive search strategy, only a small number of studies were found, which used qualitative methods to explore perspectives on the postnatal discharge process. They were conducted in diverse settings and only one included study was conducted in a lower middle-income country (Tanzania). The lack of research may be linked to the fact that coverage of postnatal care lags behind antenatal and intrapartum care and is afforded less attention in the health literature. Among the small number of included studies, the only health worker perspective that is represented is from midwives, which limits our ability to generalise the findings to nurses or other categories of health worker involved in providing care to women in the postnatal period and at discharge. Similarly, only one study considered the perspectives of fathers, which limits the generalisability of findings to other caregivers and family members aside from the mother. Our ability to generalise our findings to different types of facility is also limited because all six studies were conducted in hospital settings; the discharge process may be different and experienced differently in other types of facilities offering maternity care. Despite these limitations, this QES addresses a knowledge gap on stakeholder’s perspectives of postnatal discharge, highlighting the importance of discharge preparation as a critical opportunity to ensure the woman, parents and newborn receive support for the transition to care in the home. As the updated WHO recommendations on postnatal care^15^ become embedded in country health systems and policies, there may be renewed interest on values, preferences and perspectives at system, service and end-user level. Further qualitative research from multiple perspectives will be important to determine whether the positive effects of adequate discharge preparation (eg, enhanced well-being, confidence and experiences) are noticeable and sustained.

## Conclusion

Our QES of findings from six studies from four countries, published between 2002 and 2020, reveals an overall feeling of dissatisfaction with the current provision of discharge preparation. The perspectives of included stakeholders (women, fathers and midwives) indicate a call for increased focus on the emotional and social needs of women and families during discharge preparation, something which is often overlooked compared with the clinical assessments that take place during this period. The sense of frustration expressed by midwives and women suggests that a lack of time and resources may be the most significant barrier to ensuring quality of care prior to discharge from the facility. Although the included studies were conducted in countries which have varied policies regarding recommended length of stay at a facility after birth, the importance of timing of discharge was emphasised in most studies, as was the participation and engagement of fathers and other family members, which is critical for supporting women and caring for the newborn. Overall, the limited qualitative evidence available suggests the likely positive impact of adequate discharge preparation on the experience of care of women, men and other family members and on improving women’s and parents’ confidence to care for the baby in the home. Programme managers will need to identify and address the health service and system barriers to ensure appropriate preparation prior to discharge after birth.

## Data Availability

Data may be obtained from a third party and are not publicly available. All data included in this qualitative evidence synthesis are publicly available within the published research studies that were included in this review. The datasets themselves may be obtained from a third party and are not publicly available.

## Footnotes

Note on terminology: Throughout this paper, we have used the terms women, men, fathers, parents/caregivers and families. We recognise that not all individuals who go through childbirth identify as woman. We also recognise that diverse family structures exist. Although some of the literature included in this research focused on the experiences of fathers, we recognise that future literature and any updates of this work will need to expand these concepts.

The Swedish Ministry of Health defines discharge occurring within 3 days following birth as an early discharge.
