# Supplementary material for: Stakeholder’s perspectives of postnatal discharge: a qualitative evidence synthesis
Source: BMJ Glob Health. 2023 Aug 8;8(Suppl 2):e011766. doi: 10.1136/bmjgh-2023-011766 (PMC10414110; doi:10.1136/bmjgh-2023-011766)
Supplement: Supplementary data [file bmjgh-2023-011766supp001.pdf]

Supplementary file 1. Example search strategy for PubMed/MEDLINE (adapted for other databases)

Search string	Content
#1	Woman [mh] OR mother [mh] OR maternal [mh] OR patient [mh]
#2	Infant [mh] OR new-born [mh] OR [baby]
#3	Caregiver OR family [mh] OR partner [mh] OR husband [mh] OR parent [mh]
#4	Midwife [mh] OR nurse [mh] OR health care worker [mh] OR health care professional [mh] OR health worker [mh] OR health care provider [mh] OR physician [mh] OR paediatrician [mh] OR obstetrician [mh]
#5	#1 OR #2 OR #3 OR #4
#6	Discharge preparation [tiab] OR discharge readiness [tiab] OR readiness for discharge [tiab] OR discharge process [tiab] OR discharge OR discharge transition [tiab] OR discharge plan [tiab] OR hospital discharge [tiab] OR patient discharge [tiab]
#7	Postnatal [mh] OR postpartum [mh] OR after birth [tw] OR intrapartum [mh] OR childbirth [mh] OR maternity [mh] OR neonatal [mh]
#8	#6 AND #6 AND #7
#9	Publication year limit 2000-2020
