# Supplementary material for: Stakeholder’s perspectives of postnatal discharge: a qualitative evidence synthesis
Source: BMJ Glob Health. 2023 Aug 8;8(Suppl 2):e011766. doi: 10.1136/bmjgh-2023-011766 (PMC10414110; doi:10.1136/bmjgh-2023-011766)
Supplement: Supplementary data [file bmjgh-2023-011766supp002.pdf]

Supplementary file 2. GRADE-CERQual assessment of confidence in the findings

Studies contributing data	Synthesis/review finding	Methodological limitations	Coherence	Adequacy	Relevance	Certainty of evidence	Rationale
Dol (2019); George (2005); Haith-Cooper (2018)	Rushed discharge process	Minor concerns	Moderate concerns	Serious concerns	Moderate concerns	**Low**	Downgraded two levels for serious concerns with adequacy, moderate concerns with relevance and coherence, and minor concerns with limitations.
Dol (2019); Persson (2002); Kanotra (2007); Svensson (2018)	Diversification of teaching methods needed	Minor concerns	Moderate concerns	Moderate concerns	No or very minor concerns	**Moderate**	Downgraded one level for moderate concerns with coherence and adequacy and minor concerns with limitations.
Dol (2019); Haith-Cooper (2018)	Need for standardized and comprehensive procedures	Minor concerns	No or very minor concerns	Serious concerns	No or very minor concerns	**Moderate**	Downgraded one level for moderate concerns with adequacy and minor concerns with limitations.
Dol (2019); George (2005); Kanotra (2007); Svensson (2018)	Care for the woman is often overlooked	Moderate concerns	No or very minor concerns	Moderate concerns	No or very minor concerns	**Moderate**	Downgraded one level for moderate concerns with adequacy and moderate concerns with limitations.
Dol (2019)	Need for midwife training on postnatal care education	Minor concerns	No or very minor concerns	Serious concerns	No or very minor concerns	**Moderate**	Downgraded for serious concerns with adequacy and minor concerns with limitations.
Dol (2019); Persson (2002); Kanotra	Importance of involving fathers/other family members	Minor concerns	No or very minor concerns	Moderate concerns	No or very minor concerns	**Moderate**	Downgraded one level for moderate concerns with adequacy and minor concerns with limitations.

(2007); Svensson (2018)							
Dol (2019); George (2005)	Assumed maternal knowledge	Moderate concerns	No or very minor concerns	Serious concerns	No or very minor concerns	**Low**	Downgraded two levels for serious concerns with adequacy and moderate concerns with limitations.
Dol (2019); Haith-Cooper (2018); Kanotra (2007); Svensson (2018)	Socioeconomic, cultural and language barriers	Minor concerns	No or very minor concerns	Moderate concerns	No or very minor concerns	**Moderate**	Downgraded one level for moderate concerns with adequacy and minor concerns with limitations.
Persson (2002); Kanotra (2007); Svensson (2018)	Importance of women/parent involvement in the discharge process	Minror concerns	No or very minor concerns	Serious concerns	No or very minor concerns	**Moderate**	Downgraded one level for serious concerns with adequacy and minor concerns with limitations.
